# A systematic review on AI-driven noninvasive fetal ECG processing and analysis methods

**DOI:** 10.3389/fdgth.2026.1926578

**Published:** 2026-08-13

**Authors:** Dragos Daniel Țarălungă, Bogdan Cristian Florea, Anusuyah Subbarao, Georgeta Mihaela Neagu, Bogdan Emanuel Ionescu

**Affiliations:** 1 National University of Science and Technology Politehnica Bucharest, Bucharest, Romania; 2 Multimedia University Malaysia, Cyberjaya, Malaysia

**Keywords:** artificial intelligence, deep learning, fetal monitoring, noninvasive fetal electrocardiogram, signal processing

## Abstract

**Introduction:**

Fetal electrocardiogram is a critical tool for fetal health monitoring, and it is obtained nowadays in clinical practice through an invasive procedure. An alternative is the use of noninvasive fetal electrocardiogram (NI-fECG) that can be obtained by placing a matrix of electrodes on the maternal abdomen. However, the extraction of NI-fECG from abdominal signals (ADSs) remains challenging due to the low amplitude of fetal signals, overlapping with maternal ECG, and because of contamination from other noise sources. Traditional signal processing methods have been widely used but are often limited by incomplete separation, signal distortion, and the need for manual parameter tuning. Artificial intelligence (AI) has emerged as a powerful solution, focusing on deep learning techniques to improve NI-fECG extraction and noise suppression, offering automated feature extraction, adaptability to signal variability, and real-time processing, and addressing the limitations of classical methods. This study presents a comprehensive systematic review of AI methods in NI-fECG processing and analysis, evaluating their effectiveness and limitations.

**Methods:**

The review follows a structured methodology, including study selection and analysis of AI techniques, following PRISMA 2020 guidelines, and covering peer-reviewed studies published between 2014 and 2025. It categorizes AI models into neural network-based, generative, hybrid, and specialized architectures.

**Results:**

Twenty-two studies met the inclusion criteria. AI-based approaches outperformed traditional signal processing in NI-fECG extraction and denoising. Generative and specialized architectures showed the strongest morphology preservation, while hybrid models added robustness. Six studies applied AI to diagnostic tasks, including fetal heart rate estimation, arrhythmia detection, and congenital heart disease classification. Limitations include scarce ground-truth datasets and a lack of standardized evaluation protocols.

**Discussion:**

No single architecture is universally superior across all pipeline stages. A key research gap is the need for AI models that conserve NI-fECG morphology for medical decision-making, alongside standardized benchmarking and explainable models.

## 1 Introduction

**Figure 1:**
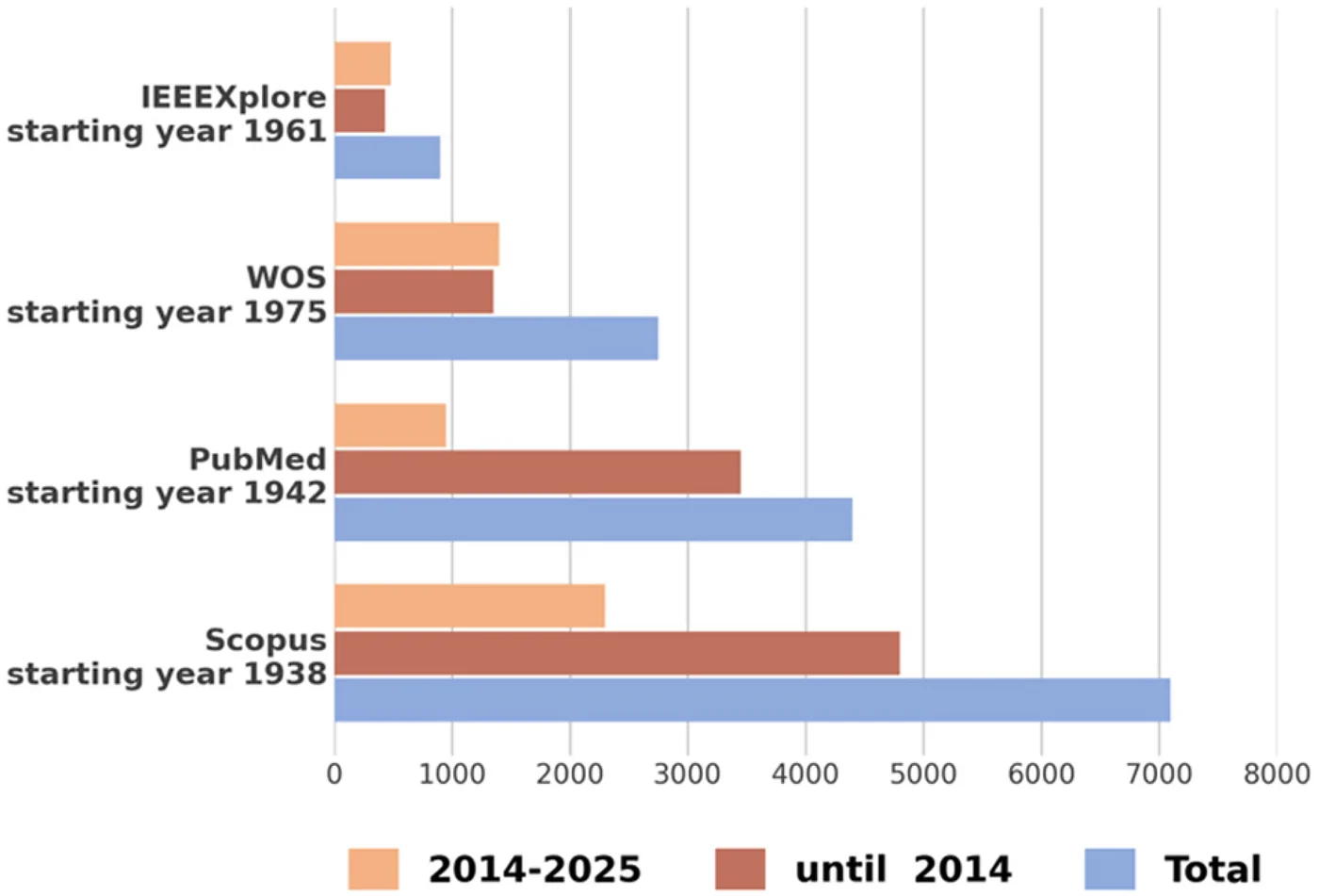
Number of articles published in the field of fECG in the main scientific datasets.

Fetal electrocardiography (fECG) as a tool for fetal monitoring has attracted the medical and biomedical engineering communities since 1938 (see Figure 1). In the last 10 years the weight of articles published in the field of fetal electrocardiography shows a significant increase in this research direction: 50.3% for WOS (starting year 1975), 50.4% for IEEEXplore (starting year 1961), 32.5% for Scopus (starting year 1938), 21.2% for PubMed (starting year 1942). This is explicable, since the fECG signal is a critical complementary tool in clinical practice used nowadays, i.e., cardiotocography, for fetal monitoring and diagnosis, providing valuable insights into fetal cardiac activity and its potential abnormalities.

Unlike conventional invasive methods, such as fetal scalp electrodes, NI-fECG enables continuous and safer monitoring by capturing electrical signals from the maternal abdomen. It is obtained by placing a matrix of electrodes on the maternal abdomen, which record a mixture of biopotentials like maternal electrocardiogram (mECG), abdominal electromyogram (abd-EMG), electrohysterogram (EHG), fECG and other signals generated by different sources. These signals are collectively referred to as abdominal signals (ADSs).

To obtain the low amplitude NI-fECG, advanced computational methods need to be employed to process the ADSs. However, significant challenges need to be overcome due to the low amplitude of fetal signals, their overlapping with maternal ECG (mECG), and contamination from various noise sources, including muscle contractions, motion artifacts and powerline interference (1, 2).

Traditional signal processing techniques have played a fundamental role in NI-fECG extraction and analysis. Methods such as adaptive filtering (3–20), blind source separation (BSS) (21–31), time-frequency analysis (32–44), different decomposition techniques like empirical mode decomposition (EMD) (45–57) and template subtraction (58–64) have been widely used to isolate fetal ECG from maternal and noise components. While these approaches have shown promising results, they face several limitations, e.g., the availability of reliable reference signal, statistical assumptions, computational complexity, application of predefined basis functions that reduce the adaptability to the signal variability in real-world conditions. Moreover, the main shortcoming of the traditional signal processing approaches is offering a NI-fECG signal which is incompletely separated from the mECG component and in some cases signal distortions are introduced. Hence, the diagnostic information encapsulated in the fECG morphology is altered and is not reliable for clinical practice. In addition, these classical methods often require manual tuning of parameters and exhibit reduced performance under low signal-to-noise ratio (SNR) conditions, which limit their robustness and generalizability.

Artificial intelligence (AI) offers a transformative solution to these challenges by proposing data-driven learning models that can automatically extract meaningful features and adapt to complex, nonlinear signal variations. AI-driven approaches, including convolutional neural networks (CNNs), recurrent neural networks (RNNs), autoencoders, and generative adversarial networks (GANs), have demonstrated superior performance in fECG extraction, noise suppression, and abnormality detection. These methods eliminate the need for manual feature engineering, adapt to the variations in fetal and maternal signals, and provide real-time processing capabilities that are essential for clinical applications. Furthermore, deep learning models can learn from large datasets, improving their accuracy over time and enabling robust performance across diverse patient populations.

Despite the lately huge progress in non-invasive fetal monitoring by investigating the ADSs collected on the abdomen of the pregnant women, the researchers still deepen the knowledge in the field, trying to better address the (morphological) analysis of the fECG, with the final aim to prevent the pre-term delivery, fetal mortality and to identify the maternal risks. The ADSs show high benefits regarding the recording procedure, since the corresponding measurement devices might be used outside the clinical institutions, being therefore suitable for (at home) long-term pregnancy monitoring (65).

The researchers have therefore focused on improving the quality of the ADSs, either by contributing to the hardware development (66) or by evaluating the quality of the ADSs and selecting the most appropriate channels, to impact the (pre)processing/classification techniques (67). The review of Liu B et al. (68) investigated the feasibility of the antenatal ambulatory fetal electrocardiography, excluding i) the studies contributing to the fetal monitoring by enhancing modelling/physiological investigations/hardware development, ii) the case and animal research and iii) the studies addressing the patient satisfaction while exposed to ADSs recordings. All the selected research works investigated within the review used the Monica AN24 device. In 2024 Liu L et al. (69) dedicated a review study to pregnancy monitoring, prioritizing the latest advancements regarding the wearable sensors, very suitable for long-term pregnancy surveillance. The research considered all the potential information that could (holistically) check the wellbeing state of the mother and fetus: the fetal/maternal cardiac activity (usually maternal ECG, maternal/fetal heart rates are provided), the uterine activity, considering both the fetal movements and the uterine contractions and the multi-modal sensor integration strategies. The corresponding processing techniques are then mentioned, as well as the strategies to establish the discriminative information necessary to early identify fetal and/or maternal complications. Nevertheless, the ADSs advanced processing and the potential fetal/maternal (AI based) diagnosis need to be still developed.

Recently a few review articles have focused on the topic of artificial intelligence and NI-fECG. Kaleem et al. (70) presents a broad overview of both classical and AI-driven techniques for fECG extraction. It examines methods such as adaptive filtering, BSS, wavelet transforms, and singular value decomposition (SVD), discussing their strengths and limitations. The study highlights the dominance of mECG signals in abdominal recordings and emphasizes the struggle of classical methods with fetal signal isolation due to overlapping QRS complexes and noise. It also briefly describes neural networks and computational intelligence techniques, highlighting the need for more robust and real-time processing approaches. In (71) the authors also include a discussion on traditional computational techniques and AI, categorizing fECG extraction methods into linear adaptive filtering, non-linear adaptive filtering including Artificial Neural Networks (ANN), Machine Learning (ML), and Deep Learning (DL) models, BSS, template subtraction, and hybrid approaches. The study also discusses a few publicly available datasets, such as PhysioNet Computing in Cardiology Challenge Dataset (PCDB) and Non-Invasive Fetal ECG Dataset (NIFECGDB). Marnani et al. (72) introduces a very general review on NI-fECG during prenatal, intrapartum and postnatal care. It explores also other diagnosis methods beside NI-fECG, electrode configuration and electrode types. The presentation and discussion on NI-fECG is limited and only a paragraph is dedicated to AI methods mentioning briefly just three approaches. Similarly, Li et al. (73) and Pégoire et al. (74) adopt a literature view of fetal monitoring technologies, including cardiotocography, photoplethysmography, and NI-fECG among others. These reviews present fECG as one of several physiological signals of interest and mention briefly AI as potentially improving the NI-fECG processing and analysis. More recently, Huang et al. (75) realized a survey focused only on the deep learning approaches for fetal ECG extraction. However, compared to existing review articles that predominantly focus on deep learning techniques for fetal ECG extraction (75), the present study provides a broader and more comprehensive perspective by systematically analyzing the entire AI-driven NI-fECG processing pipeline, including extraction, denoising, and diagnostic analysis. While prior works mainly emphasize detection performance, particularly fetal QRS identification, our review extends the evaluation toward clinically relevant aspects, such as waveform morphology preservation and diagnostic fidelity. Furthermore, this study introduces a structured taxonomy that encompasses neural network-based, generative, hybrid, and specialized architectures, enabling a more coherent comparison across heterogeneous approaches. In addition to methodological analysis, we critically examine dataset limitations, generalization issues, and the lack of standardized benchmarking frameworks, aspects that remain insufficiently addressed in previous literature. By explicitly linking AI performance to clinical usability and highlighting gaps related to explainability, data availability, and multi-task learning, this review offers a more application-oriented synthesis. Consequently, it remains highly relevant, as it not only consolidates recent advances but also provides clear directions for developing robust, clinically applicable AI solutions for noninvasive fetal monitoring.

The present review follows a rigorous methodology, including the selection of relevant studies, defining research questions, analyzing the identified AI techniques, comparing their performance metrics, and identifying key challenges, literature gaps and future research directions. Our findings will contribute to the growing body of knowledge on AI-driven NI-fECG processing and analysis, supporting researchers and clinicians in developing more accurate and efficient diagnostic tools for fetal health assessment. The paper is structured in 5 sections: i) Introduction, ii) Methodology that includes the review protocol, the search strategy, study selection, data extraction and quality assessment, iii) Datasets that consists of a detailed presentation of all fECG datasets, both simulated and real signals, iv) Performance Metrics which aims to identify the most used evaluations parameters of the AI approaches, v) Section 5 which emphasize the included AI approaches, their specific strategies to preprocess and organize the input data, and detailing their architectures and results iv) Discussions that focus on the comprehensive and critical assessment of the performances and limitations of the identified AI-driven approaches, on formulating answers to the defined research questions, on identifying literature gaps and on proposing future critical directions for a successful integration of AI in NI-fECG preprocessing and analysis that will ensure its use in clinical practice fetal monitoring; vi) Conclusions.

## 2 Methodology

The purpose of this literature review is to identify, collect, and analyze existing literature to answer frontier predefined research questions. The protocol we define ensures transparency, consistency, and replicability throughout the review process.

**Figure 2:**
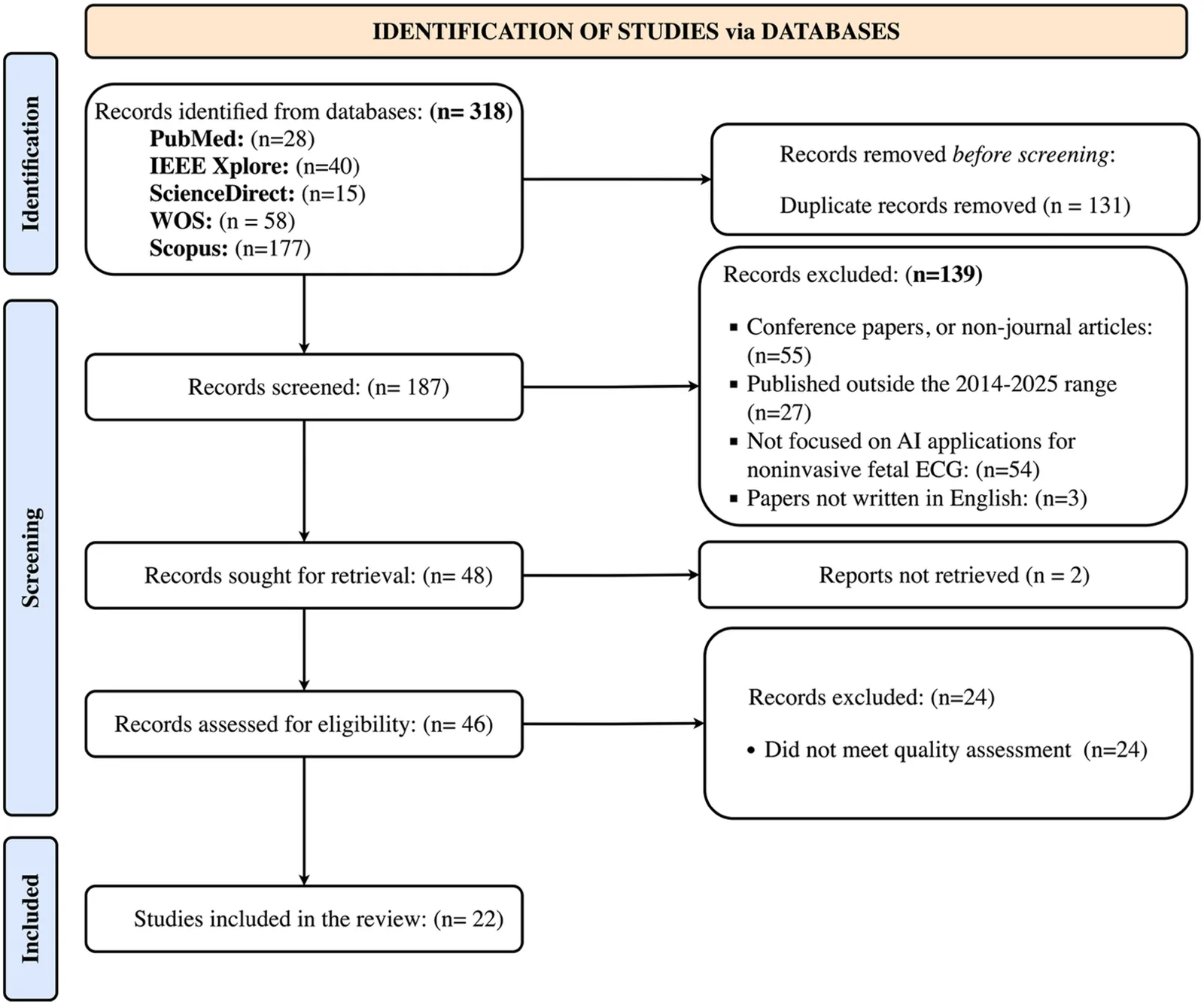
PRISMA flow diagram.

The primary objective of this review is to analyze the current challenges and advancements in using AI for NI-fECG processing and analysis. Specifically, the proposed review aims to identify key methodologies and frameworks used in the domain of processing and analysis of NI-fECG signals, assess the performance of applied AI models, highlight research gaps and future directions. PRISMA (Preferred Reporting Items for Systematic reviews and Meta-Analyses and network meta-analyses) reporting guidelines is followed throughout (see Figure 2) (76).

### 2.1 Research questions

The formulation of the research questions (RQs) is guided by the need to systematically evaluate the role and effectiveness of AI methods in the NI-fECG signal processing and analysis pipeline. In alignment with the PRISMA framework, we followed a top-down approach to define questions that reflect the entire analytical pipeline—from data availability to diagnostic decision support.

The key RQs guiding this review are:

RQ1: What are the characteristics and availability of the fECG datasets used for AI models?

RQ2: Do AI approaches used for NI-fECG improve the outcome of fetal monitoring?

RQ3: Which AI model is the most appropriate to process and analyze the NI-fECG signal for medical decision- making?

RQ4: What are the current challenges of AI models for processing the NI-fECG signal?

RQ1 focuses on understanding the characteristics, accessibility, and diversity of datasets used for AI training and validation, as high-quality and standardized data are foundational for robust model development. RQ2 addresses the direct impact of AI techniques on improving various stages of the NI-fECG pipeline, such as signal extraction, denoising, and feature interpretation. RQ3 aims to identify the most suitable AI models for NI-fECG processing and analysis, taking into account performance, explainability, and potential clinical integration. Finally, RQ4 highlights current limitations and bottlenecks that hinder the full deployment of AI in this domain. These RQs are refined iteratively based on initial exploratory searches and gaps observed in existing reviews. Their structured formulation ensures that the review comprehensively covers methodological trends, practical implications, and future research directions.

### 2.2 Search strategy

A structured search query is created using relevant keywords and Boolean operators:

*((TI* *=* *[(“artificial intelligence” OR “AI” OR “machine learning” OR “deep learning” OR “artificial neural networks”) AND (“fetal electrocardiogram” OR “fetal ECG” OR “fetal electrocardiography”)]) OR AB* *=* *[(“artificial intelligence” OR “AI” OR “machine learning” OR “deep learning” OR “artificial neural networks”) AND (“fetal electrocardiogram” OR “fetal ECG” OR “fetal electrocardiography”)]) OR AK* *=* *[(“artificial intelligence” OR “AI” OR “machine learning” OR “deep learning” OR “artificial neural networks”) AND (“fetal electrocardiogram” OR “fetal ECG” OR “fetal electrocardiography”)]*, where TI stands for “Title”, AB for “Abstract” and AK for “Authors Keywords”. The datasets searched was Web of Science, ScienceDirect, IEEEXplore, PubMed and Scopus.

The inclusion criteria for our literature review are: 1. Only paper from 2014 onwards (until 1.03.2025 when the review process started), 2. Studies focusing on AI applications for NI-fECG processing, 3. Papers that discuss model performance, datasets, and validation techniques, 4. Articles published in peer-reviewed journals.

The exclusion criteria are defined as following: 1. Studies focused solely on invasive fECG techniques (direct fECG signal obtained by placing an electrode on the fetus scalp), 2. Non-AI-based fECG studies (e.g., traditional signal processing only), 3. Papers not written in English., 4. Conference papers.

We have introduced a review-specific quality assessment checklist to evaluate the methodological quality and reporting completeness of the included studies. The checklist consist of six criteria addressing study relevance, AI methodology, dataset and performance reporting, comparison with conventional methods, validation strategy, and discussion of limitations (Table 1). Each criterion was scored on a three-point scale (1 = Poor, 2 = Fair, 3 = Excellent), resulting in a maximum possible score of 18. To ensure the inclusion of studies with adequate methodological quality, a minimum score of 15 out of 18 is required. This threshold is selected to exclude studies with substantial methodological or reporting limitations while retaining studies that provided sufficient detail for meaningful analysis and comparison. The quality assessment was performed independently by two independent reviewers. Any disagreements were resolved through discussion until consensus was reached.

**Table 1:** Quality assessment criteria.

Code	Quality criteria
QA1	Is the study relevant to AI in fECG processing and analysis?
QA2	Does the study clearly describe the AI methodology?
QA3	Are performance metrics and datasets explicitly stated?
QA4	Does the study compare AI methods to traditional techniques?
QA5	Is the validation method (clinical or simulation-based) adequate?
QA6	Are limitations and future research directions discussed?

## 3 Results

This section presents a comprehensive presentation of all datasets that are usually used when evaluating computational methods for NI-fECG processing and analysis, of preprocessing phase, the input data organization and the architecture of the AI models.

### 3.1 Simulated NI-fECG datasets

Several studies included in this review rely on simulated abdominal ECG signals generated using mainly the FECGSYN toolbox, a well-established open-source framework. This tool models the maternal and fetal hearts as electrical dipoles with configurable spatial positions and magnitudes, allowing simulation of diverse physiological scenarios. Each component of the abdominal signal, mECG, fECG, and noise, is generated as an independent signal source, allowing to fully configure its characteristics.

The most widely used simulation setup is Dataset 2 (Table 2), introduced in (78), which models maternal and fetal dipoles under seven physiological scenarios: baseline (with/without noise), fetal movement, heart rate variations, uterine contractions, ectopic beats, and twin pregnancy. Muscular noise is added at SNRs of 0–12 dB, yielding 1,750 ADSs via dipole permutations.

**Table 2:** Simulated NI-fECG datasets.

Code	Privacy	Simulated signals	Total no. of ADSs	Length	Observations
Dataset 1 (77)	Private	32 ADSs channels 2 mECG channels	70,000		Sampling frequency (Fs): 250 Hz Three different physiological cases were simulated: Case 0: Baseline with noise Case 1: Fetal movement with noise Case 2: Maternal and fetal heart rate acceleration/deceleration with noise SNR of fECG over noise: 0 dB, 3 dB, 6 dB, 9 dB, 12 dB, 15 dB.
Dataset 2 (78)	Public	32 simulated ADSs channels 2 simulated mECG channels	1,750	5 min	Fs: 250 Hz Seven different physiological events were taken into consideration for simulating synthetic signals Noise source: muscular noise with signal-to-noise ratios (SNR) of 0, 3, 6, 9, and 12 dB
Dataset 3 (79)	Private	32 simulated ADS channels 2 simulated mECG channels			Fs: 250 Hz random modifications to the available VCGs are applied in order to increase the variability of the dataset.
Dataset 4 (80)	Private				the simulated fECG signals are derived from real adult ECG signals from public datasets: PTB Diagnostic ECG Dataset, QT Dataset, St.-Petersburg Institute of Cardiological Technics 12-lead Arrhythmia Dataset
Dataset 5 (81)	Private		40,000		Fs: 250 Hz the simulated fECG signals are derived from real adult ECG signals from public datasets: MIT-BIH Normal Sinus Rhythm (NSRDB), Fantasia Dataset, MIT-BIH ST Change Dataset (STDB)

Dataset 1 was generated with the same simulator but only three scenarios (Cases 0–2), segmented by SINR into high (20,000), medium (20,000), and low quality (10,000), with added artifacts such as baseline wander and electrode motion. Including 20,000 validation segments, it totals 70,000 ADSs (76).

Fotiadou et al. (79) expanded Dataset 2 into Dataset 3 by introducing randomized intervals and amplitude modulations of PQRST complexes, enhancing morphological diversity for training robustness. Dataset 4 (80) adapted real adult ECGs (PTB, QT, St. Petersburg datasets), applying high-pass filtering, smoothing, and time-scaling to match fetal heart rate characteristics, then mixing with abdominal noise for realism.

Finally, Dataset 5 (81) combined adult ECGs (MIT-BIH NSRDB, Fantasia, MIT-BIH STDB) as maternal signals with synthetic fECG from Dataset 2, producing 40,000 ADSs (∼300,000 beats) with amplitude scaling to mimic SNR variability.

### 3.2 Real NI-fECG datasets

A total of 15 real-world abdominal ECG signal datasets were identified across the reviewed studies. These datasets vary in terms of availability (public vs. private), recording configurations, number of subjects, length and type of recordings, and whether they include reference signals such as direct fetal ECG (fECG) obtained via invasive scalp electrodes.

A summary of their characteristics, including the type of signals, number of recordings, sampling frequencies, and availability of ground truth fECG, is provided in Table 3.

**Table 3:** Real NI-fECG datasets.

Code	Privacy	Signals recorded	No. of subjects/ADSs	Length	Observations
Dataset 6 (67)	Private	24 unipolar ADSs channels	55/170		GS: 21–27 weeks Fs: 2,048 Hz/resampled at 500 Hz high-pass Butterworth filter, 1 Hz. Fetal R-peaks were manually annotated
Dataset 7 (81)	Public	24 unipolar ADSs channels 3 thorax ECG channels 1 maternal respiration channel fetal pulsed-wave Doppler	39/1,440	7.5 s to 119.8 s	GS: 21–27 weeks Fs: 2,048 Hz Bandwidth: 550 Hz
Dataset 8 (78)	Public	4 ADSs channelsx	75/300	1 min.	GS: not mentioned Fs: 1,000 Hz The recordings are obtained from multiple sources using a variety of instrumentation
Dataset 9 (82)	Public	4 ADSs channels 1 direct fECG channel	5/20	5 min	GS: 38 – 41weeks Fs: 1,000 Hz Bandwidth 1–150 Hz, R-peak locations of fECG annotated by experts
Dataset 10	Public	5 ADSs channels 3 mECG channel	1/5	10 s	GS: not mentioned Fs: 250 Hz
Dataset 11 B1 (83)	Private	4 ADSs channels	10/40	20 min	GS: 32 – 42weeks Fs: 500 Hz Annotations: 28,405 fetal QRS and 18,936 maternal QRS complexes
Dataset 12 B2 (83)	Partially public	4 ADSs channels 1 direct fECG channel	12/48	5 min	GS: 38 – 42weeks Fs: 500 Hz for ADSs channels and 1 kHz for the direct fECG channel. Annotations: 7,903 fetal QRS and 5,177 maternal QRS complexes Only 5 records are made public.
Dataset 13 (84)	Private	12 ADSs channels	190/2,280	20 min	GS: 19–40 weeks Fs: 1,000 Hz Initially the dataset contained 406 subjects. After excluding those with missing information or abnormal conditions 190 subjects where kept.
Dataset 14 (85)	Private	1 ADSs channel	28/28		GS: 36–42 weeks Fs: 500 Hz The total duration of the dataset is approximately 91 h
Dataset 15 (86)	Public	3/4 ADSs channels 2 thoracic mECG channels	1/55	variable	GS: 21–40 weeks Fs: 1,000 Hz Bandwidth: 0.01 Hz–100 Hz Resolution: 16 bits Annotation for QRS complexes.
Dataset 16 (87)	Public	4/5 ADSs channels 1 thoracic mECG channel	26/129	≈ 10 min	GS: not mentioned Fs: 500 Hz/1,000 Hz For 25 subjects there are 5 ADSs channels and for one subject there are 4 12 subjects presented fetal arrhythmia and 14 subjects (control) without arrythmia.
Dataset 17 (88)	Private	6 ADSs channels	-/462	variable (from 5 to 50 min)	GS: 18–24 weeks Fs: 500 Hz additional two electrodes were used as a common reference and ground, placed close to the navel ADSs channels no. 1, 3, 4 and 5 offer optimal signal clarity and spatial coverage The ADSs are preprocessed and the mECG is eliminated
Dataset 18 (89)	Public	1 ADSs channel 1 direct fECG signal 1 thoracic mECG	–/12	5 min	GS: not mentioned Fs 1,000 Hz for ADSs channels and 500 Hz for the mECG channel
Dataset 19 (90)	Private	6 ADSs channels	–/451	≈30 min	GS: 18–24 (healthy), 18–30 (CHD) weeks Fs 500 Hz the maternal ECG is removed from ADSs in the preprocessing stage.
Dataset 20 (91)	Private	12 ADSs channels 1 thoracic mECG	70/840		GS: 18 – 42weeks Fs 1 kHz 0.05– 100 Hz bandpass filtering

#### 3.2.1 Performance metrics

An important discussion topic refers to how the performance of the AI approaches is evaluated in the context of fECG processing and analysis. It depends in which stage of the pipeline for NI-fECG signal processing and analysis the AI is used. Thus, the AI methods used for fECG extraction from ADSs or fECG denoising, have as primary target to obtain a clean fECG signal, with no morphological disturbances. We have identified two main evaluation approaches: one is based on fQRS/R-peak detection for the extracted/denoised NI-fECG (92), and the other one evaluates how well is the NI-fECG morphology is preserved after extraction/denoising.

For R-peak detection evaluation performances, the labels for R peak, usually present in all datasets, are used to determine classical performance indices which are computed as shown in Equations 1–5:

$$\begin{array}{c} Acc=\;\frac{TP+TN}{TP+TN\;+\;FP+FN}, \end{array}$$ (1)

$$\begin{array}{c} PPV=\;\frac{TP}{TP+FP},\; \end{array}$$ (2)

$$\begin{array}{c} SE\;=\;\frac{TP}{TP+FN}, \end{array}$$ (3)

$$\begin{array}{c} SP\;=\;\frac{TN}{TN+FP}, \end{array}$$ (4)

$$\begin{array}{c} \;F_{1}score\;=\;2*\frac{PPV*SE}{PPV+SE}, \end{array}$$ (5)

where PPV is the positive predictive value (or Precision), SE is sensitivity (or Recall or true positive rate), SP is specificity (or true negative rate), F1 is the F1 score (or F score or F measure or dice similarity coefficient) N is the total number of the reference R peaks, TP is the number of true positive detections (correctly identified R peaks), FP is the number of false-positive detections (non R peaks detected as R peaks), FN is the number of false-negative detections (missed R peaks).

For evaluating how well the morphological aspects of the NI-fECG signal are conserved after extraction/denoising, there are many performance indices that can be computed. In Table 4 is presented a summary of all the performance indices used in the articles included in this review.

**Table 4:** Performance parameters to evaluate the preservation of NI-fECG signal's morphology.

Parameter	Mathematical expression	Parameter	Mathematical expression
Pearson Correlation Coefficient	$\begin{array}{c} PCC\;=\;\frac{\sum (\;fECG_{p}-{\bar{\;fECG}}_{p})(\;fECG_{gt}-{\bar{\;fECG}}_{gt})}{\sqrt{\sum {\left(\;fECG_{p}-{\bar{\;fECG}}_{p}\right)}^{2}\sum {\left(\;fECG_{gt}-{\bar{\;fECG}}_{gt}\right)}^{2}}}, \end{array}$ (6)	Peak Signal-to-Noise Ratio	$\begin{array}{c} PSNR\;=\;20\;\log_{10}\frac{\;fECG_{max}}{||\;fECG_{gt}-fECG_{p}|{|}_{2}^{2}},\; \end{array}$ (7)
Spectral correlation score	$\begin{array}{c} \eta_{spec}=1-\;\;\frac{1-PCC(PSD(\;fECG_{gt}),PSD(\bar{\;fECG_{gt}}))}{PCC(PSD(\;fECG_{gt}),PSD(ADS))}, \end{array}$ (8)	The determination coefficient	$\begin{array}{c} R^{2}=1-\frac{{\left(\;fECG_{gt}-fECG_{p}\right)}^{2}}{{\left(\;fECG_{gt}-\;{\bar{\;fECG}}_{gt}\right)}^{2}}, \end{array}$ (9)
Mean Absolute Error	$\begin{array}{c} MAE\;=\;\frac{\sum_{i=1}^{n}|\;fECG_{p}-fECG_{gt}|}{N},\; \end{array}$ (10)	Signal-to-Noise Ratio improvement	$\begin{array}{c} SNR_{imp}=SNR_{in}-\;SNR_{out}, \end{array}$ (11) $\begin{array}{c} SNR_{in}=10*\log_{10}\left(\frac{\sum_{n=1}^{N}fECG_{gt}^{2}}{\sum_{n=1}^{N}{\left({\hat{\;fECG}}_{gt}-fECG_{gt}\right)}^{2}}\right),\; \end{array}$ (12) $\begin{array}{c} SNR_{in}=10*\log_{10}\left(\frac{\sum_{n=1}^{N}fECG_{gt}^{2}}{\sum_{n=1}^{N}{\left({\hat{\;fECG}}_{gt}-fECG_{gt}\right)}^{2}}\right),\; \end{array}$ (13)
Root Mean Squared Error	$\begin{array}{c} RMSE\;=\;\sqrt{\frac{\sum_{i=1}^{n}{\left(\;fECG_{gt}-fECG_{p}\right)}^{2}}{N}}, \end{array}$ (14)	Percent-Root Distortion	$\begin{array}{c} PRD=100*\sqrt{\frac{\sum_{n=1}^{N}{\left(\;fECG_{gt}-\;fECG_{p}\right)}^{2}}{\sum_{n=1}^{N}fECG_{gt}^{2}}},\; \end{array}$ (15)
Structural Similarity Index	$\begin{array}{c} SSIM\;=\;\frac{\left(2{\bar{\;fECG}}_{gt}{\bar{\;fECG}}_{p}+c_{1})(2\sigma_{\;fECG_{gt}fECG_{p}}+c_{2}\right)}{\left({\bar{\;fECG}}_{gt}^{2}+{\bar{\;fECG}}_{p}^{2}+c_{1})+(\sigma_{\;fECG_{gt}}^{2}+\sigma_{\;fECG_{p}}^{2}+c_{2}\right)}, \end{array}$ (16)		

where *N* is the total number of data samples, *PSD is the power spectral distribution,* $fECG_{p}$ is the predicted (extracted) fECG signal, $fECG_{gt}$ is the ground truth fECG signal, ${\bar{\;fECG}}_{p}$ is the mean value of the predictions and ${\bar{\;fECG}}_{gt}$ the mean value of the true values, $\sigma_{\;fECG_{gt}}^{2}$ is the variance of the $fECG_{gt}$, $\sigma_{\;fECG_{p}}^{2}$ is the variance of the $fECG_{p}$, $\sigma_{\;fECG_{gt}fECG_{p}}$ is the covariance of the $fECG_{gt}$ and $fECG_{p}$, while the dynamic range of data is modeled with the constants $c_{1}$ and $c_{2}$, ${\hat{\;fECG}}_{gt}$ the noisy version of the $fECG_{gt}$.

### 3.3 AI computational methods for NI-fECG processing and analysis

AI techniques have emerged as promising tools to address critical challenges in utilizing NI-fECG for fetal health assessment: i) estimate a clean NI-fECG signal from ADSs, i.e., free from maternal and environmental interference, without compromising the diagnostic integrity of its morphological features and ii) to use the diagnostic information encapsulated in the estimated NI-fECG signal, for evaluating the fetal health.

**Figure 3:**
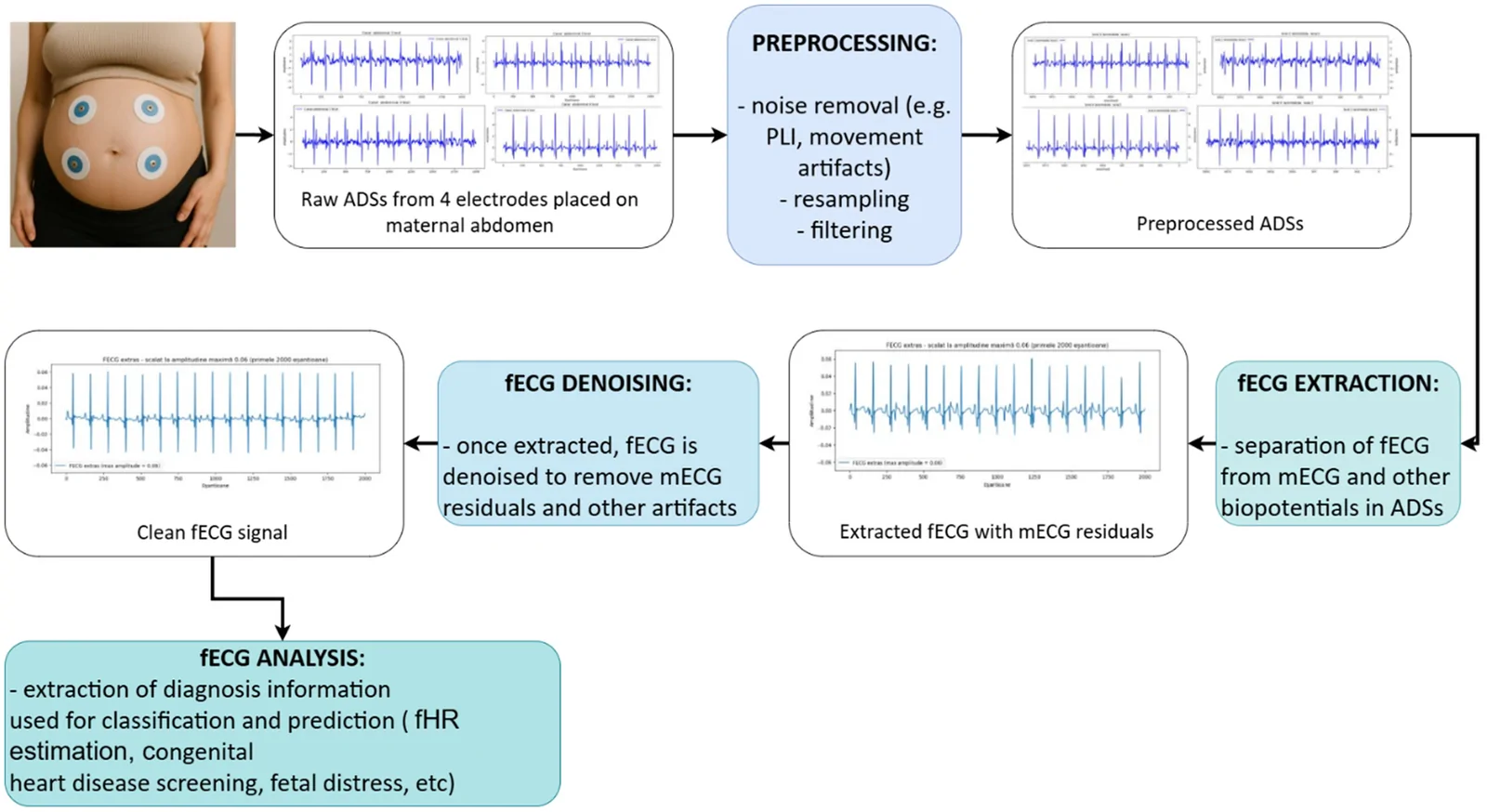
The pipeline for NI-fECG signal processing and analysis.

The reviewed literature demonstrates that AI is applied across three key stages of the NI-fECG processing and analysis pipeline (illustrated in Figure 3): (i) NI-fECG extraction: Separation of the fetal ECG component from the maternal ECG (mECG) and other biopotentials and noise components that are part of the ADSs, (ii) NI-fECG denoising: Post-extraction enhancement of the NI-fECG signal to suppress residual maternal or ambient noise, thereby improving signal quality for diagnostic interpretation, (iii) NI-fECG analysis for diagnostic: Interpretation of the cleaned NI-fECG signal to detect and classify fetal cardiac anomalies or arrhythmic patterns.

The AI methods covered in this review are categorized and analyzed based on their primary contribution to one or more of these three processing stages.

The diagnostic information in the NI-fECG signals refers to the amplitude, duration, and waveform morphology of clinically relevant components such as the P, Q, R, S, and T waves, as well as the PR, ST segments, and PR, RR, and QT intervals.

To systematically analyze the AI-based NI-fECG studies, we classify the methods into four categories based on their architecture and methodological focus:

- **Neural Network–Based Approaches**—general-purpose architectures such as CNNs, RNNs, and their variants, commonly applied for pattern recognition, classification, or feature extraction in NI-fECG data.
- **Generative Models**—approaches like GANs and Variational Autoencoders (VAEs) that learn the underlying data distribution to synthesize, augment, or enhance signals, often used for realistic signal simulation or denoising.
- **Hybrid Models**—methods combining deep learning with traditional signal processing or optimization techniques, e.g., integrating ICA or wavelet transforms with neural networks, or pairing handcrafted features with classifiers such as SVM (Support Vector Machine) or Random Forests.
- **Specialized Architectures**—NI-fECG–specific designs incorporating domain knowledge, custom layers, or task-specific loss functions, including attention-guided networks, fetal-focused U-Nets, and tailored encoder–decoder models for fECG extraction or analysis.

Further, the discussion of the included AI approaches is organized according to the 3 main contribution that they can be used for NI-fECG processing and analysis, mentioning for each, their category, their characteristics and performances reported.

Further, the discussion of the included AI approaches is organized according to the 3 main contribution that they can be used for NI-fECG, mentioning for each their category, their characteristics and performances reported.

#### 3.3.1 AI methods for fECG extraction

A significant number of the studies included in this review focus on the extraction of the NI-fECG signal ADSs, which is the first and most critical step in NI-fECG analysis. This process typically involves separating the fECG from the dominant maternal ECG (mECG) and noise components.

##### 3.3.1.1 Neural network-based approaches

Several studies employ deep learning architectures like CNNs and RNNs for fECG extraction from abdominal recordings. Zhong et al. (80) trained a deep learning-based residual convolutional encoder-decoder network (RCED-Net) trained on signals generated using FECGSYNDB (Dataset 2), simulating a variety of SNR conditions. Five convolutional layers extract low- and high-level features related to the fECG and mECG components that are present in the ADSs input. The input is a 1 × 1,000 segment of ADS, representing 4 s of signal resampled at 250 Hz which previously is preprocessed. Each convolutional layer has 64 filters of size 1 × 3, with zero padding to maintain the input dimensions. The extracted features are passed through five deconvolutional layers to reconstruct the fECG signal. The final output is generated through a fully connected layer, producing the predicted fECG of size 1 × 1,000.

Vo et al. (93) introduce an end-to-end deep learning model that combines CNNs and RNNs. Firstly, 1-D Octave Convolution (1-D OctConv) is used for feature extractions. Thus, each input window is processed through a series of 1-D octave convolution layers. High-frequency features capture details like QRS spikes, while low-frequency features provide global signal trends. Inter-frequency communication ensures integrated feature learning. The extracted features flow through three residual blocks with skip connections, enabling deep feature extraction while preserving gradient stability. Bi-directional gated recurrent units (GRUs) are used to capture sequential dependencies and context over time and to ensure the model considers temporal relationships when identifying QRS complexes. Thus, the model can recognize sequential patterns in the ECG data, which is essential for detecting QRS complexes accurately. To enhance interpretability, the model employs a gradient-weighted class activation mapping (Grad-CAM) method adapted to 1-D time series. This technique generates attention maps highlighting the most discriminative regions in the ECG signal. A SoftMax layer predicts the presence or absence of QRS complexes and finally, predictions are aggregated across time and detected QRS complexes are validated against annotated references.

In 2025, Hsu et al. (97) proposed deep learning architecture, MEG-Net (Multi-scale Encoder Gated Network), for NI-fECG extraction. MEG-Net simultaneously receives mECG and ADSs inputs, each formatted as 1 × 1,024 sequences, and aims to regress the output toward the ground-truth fECG waveform. The architecture includes dual encoder branches, each comprising two convolutional layers with kernel sizes 5 and 7, respectively. Starting with 16 kernels, the number of filters doubles at each of the six spatial levels until reaching a bottleneck with 1 × 32 latent features. Batch normalization and GeLU activation functions are applied after each convolution. In the bottleneck, the latent mECG representation is subtracted from the ADSs, and the resulting difference passes through a Tanh activation to model non-linearities. The decoder path mirrors the encoder with transposed convolutional layers, skip connections, batch normalization, and GeLU activation to reconstruct the fECG waveform. MEG-Net was evaluated on simulated Dataset 2 and real ADSs (Dataset 8 and Dataset 9) obtaining the best metrics (see Table 5) on Dataset 2 and Dataset 9 when compared to classical signal processing and other AI approaches reported in literature. However, the performance on Dataset 8 is lower than classical methods and other AI architecture.

**Table 5:** Classification, characteristics and performances of the AI models used for NI-fECG extraction.

Included Study	Dataset	Input data characteristics	Performance evaluation	
			R-peak detection	Morphology conservation
Neural Network based models				
Zhong et al. (80)	Dataset 2 Dataset 8 Dataset 9	− Dataset 2 −60,000 segments (one segment has 1 × 1,000 sample) for training. − Dataset 8 and Dataset 9–2.475 segments to evaluate the model performance.	Dataset 8: SE 96.06% PPV 92.25%, F1 94.1% Dataset 9: SE 92.6%, PPV 94.68%, F1 93.62	Not performed
Vo et al. (93)	Dataset 8	− matrices of 4 × 1,000 (1 s length segments). − further segmentation: each segment is divided into 10 frames of 100 samples. − each frame is labeled as Class 1 (if it contained an annotated fQRS complex) or Class 0 (otherwise).	SE 90.32%, PPV 91.82%, F1 91.1%	Not performed
Hsu et al. (97)	Dataset 2 Dataset 8 Dataset 9	− 1 × 1,024 size inputs for ADSs, mECG and fECG ground truths. − Training set: first 7 subjects from Dataset 2. − Testing: Dataset 2 (last 3 subjects), Dataset 8 and Dataset 9	Dataset 2: Acc 89.58%, SE 91.96%, PPV 95.15%, F1 92.81% Dataset 8: Acc 73.29%, SE 76.19%, PPV 80.81%, F1 78.18% Dataset 9: Acc 97.3%, SE 98.36%, PPV 98.62%, F1 98.62%	Dataset 2: PCC 0.94, SSIM 0.96, PSNR 37.03
Generative Models				
Basak et al. (99)	Dataset 9 Dataset 11	− *N x M x C* matrix, N—number of signal segments, M—length of each segment (512 samples), *C*—number of channels. − 5-Fold cross-validation strategy. − Training: Dataset 9 and Dataset 11 B1. − Testing: Dataset 11 B2.	Acc 92.6%, SE 94.8%, PPV 97.6%, F1 96.4%	PCC score—88.4%, MAE—0.071, RMSE is 0.107, Spectral correlation score 89.4%
Qu et al. (101)	Dataset 8 Dataset 9	− 1 × 200 samples ADSs segments. − 5 fold cross-validation	Dataset 8: SE 99.43%, PPV 99.56%, F1 99.50%, Dataset 9 SE 98.6%, PPV 98.24%, F1 98.42%	PCC 0.91, SSIM95.54, PSNR 38.87, MSE 0.036, MAE 0.009
Yang et al. (102)	Dataset 2 Dataset 8 Dataset 9	− 1 × 1,024 ADSs segments. − Training set: 27,104 segments. − Test set: 3,848 segments	Dataset 9:SE 99.34%, PPV 99.31%, F1 99.93%	Dataset 9: MSE 0.019, MAE 0.006, R^2^ 98.01%
Hybrid Models				
Baldazzi et al. (67)	Dataset 6 Dataset 7	− Extracted features: spectral power, morphological characteristics (amplitude and shape features relevant to fECG signals) and fetal SNR. − Training: 10,336 segments. − Balanced dataset.	Acc 88.2%, SE 88.4%, PPV 84.6%, F1 86.5% SP 84%	Not performed
Ziani et al. (103)	Dataset 10	− images, TFIs and TSIs, generated from the ADSs using SVD, ICA, CWT, NMF, EMD. − Training and test sets: not mentioned	Acc 95%	Not performed
Specialized Architectures				
Almadani et al. (107)	Dataset 2 Dataset 8 Dataset 9	− Segments of 1 × 992 samples. − Training: Dataset 2 (4 out of 10 subjects). − Test: Dataset 2 one subject, Dataset 8 and Dataset 9.	Dataset 8: Se 98.12%, PPV 98.6%, F1 98.9%. Dataset 9: SE 99.45%, PPV 99.5%, F1 99.88%	Dataset 2: the mean PSNR 51.8 dB and SSIM 0.96 Dataset 9: the mean PSNR 31.1—41.6 dB and SSIM 0.88–0.98
Rahman et al. *(*106)	Dataset 9 Dataset 11	− *NxMxC* matrix, where *N*—number of signal segments, *M*—segment length (512 samples) and *C*—number of channels (4 channels for the ADSs). − The ground truth, the direct fECG signal is structured similarly, *NxMx1*	Dataset 9: SE 99.5%, PPV 99.7%, F1 99.6% Dataset 11 (B1): SE 99.6%PPV 99.4%, F1 99.5% Dataset 11 (B2): SE 99.3%PPV 99.5%, F1 99.4%	Dataset 9: MAE 0.068, RMSE 0.089, PCC 85.58% Dataset 11 (B1): MAE 0.061, RMSE 0.085, PCC 87.60%
Wang et al. (89)	Dataset 9 Dataset 11 (B2)	− 4 × 400 matrice of ADSs segments. − Training: 5-fold and 12-fold cross-validation.	Dataset 9: SE 99.4%PPV 99.61%, F1 99.56%	Dataset 9: MSE 0.057, MAE 0.127 Dataset 11 (B2): MSE 0.067, MAE 0.179
LEE et al. (105)	Dataset 2 Dataset 8 Dataset 9	− Segments of 1 × 1,024. − Training: Dataset 2 (ADSs 1, 8, 11, 19, 22, 25 and 32). − Test: Dataset 2 (one subject), Dataset 8 and Dataset 9.	Dataset 8: Se 95.74%, PPV 97.45%, F1 96.91%. Dataset 9: SE 98.23%, PPV 99.3%, F1 98.81%	Not performed
Rasti et al. (77)	Dataset 1 Dataset 8	− Segments of 1 × 1m024	SE 93.52%, PPV 97.4%, F1 95.42%	R^2^ coefficient is almost 1 (100%), being obtained for the highest (SNR)

##### 3.3.1.2 Generative models

Basak et al. (99) proposed a deep generative model for fetal ECG extraction based on a one-dimensional Cycle-Consistent Generative Adversarial Network (1D CycleGAN), categorized under Generative Models. Their architecture consists of two generators that perform bidirectional mapping between abdominal ECG signals and fetal ECG (fECG) signals, as well as two discriminators trained to distinguish real from generated signals. The generator networks are constructed using 1D convolutional layers and residual blocks, and uniquely apply a sine activation function to preserve ECG waveform morphology. To ensure both fidelity and realism in the extracted signals, the training process incorporates three key loss functions: adversarial loss (to encourage realistic signal generation), cycle-consistency loss (to maintain accurate bidirectional mappings), and identity loss (to preserve features when no transformation is required). The model was trained over 150 epochs with a learning rate of 0.00001, and ablation studies were conducted to fine-tune architectural parameters. Quantitative evaluation demonstrated strong fQRS detection performance, achieving an F1-score of 96.4%, accuracy of 92.6%, and sensitivity of 94.8%(Table 5). More importantly, the study also assessed how well the extracted signals retained diagnostic information. Morphological evaluation showed high waveform fidelity, with a mean absolute error (MAE) of 0.0043, root mean square error (RMSE) of 0.032, peak signal-to-noise ratio (PSNR) of 38.6 dB, and structural similarity index (SSIM) of 0.997 (Table 5). These metrics indicate a strong preservation of fECG morphology, essential for downstream diagnostic interpretation and clinical reliability.

Qu et al. (101) propose an enhanced CycleGAN-based model for NI-fECG extraction. It incorporates a dual attention mechanism: a Multi-Scale Attention (MSA) module, which filters relevant temporal features, and a Channel Self-Attention (CSA) module, which highlights key inter-channel dependencies. These modules improve suppression of maternal ECG interference and enhance fetal signal clarity. A triplet contrastive loss is added to the adversarial objective to align the generated fECG with the ground truth (direct fECG) references. Instance normalization and sine activation functions are used throughout to stabilize training and preserve morphology. Evaluation on Dataset 8 and Dataset 9, demonstrates strong performance, with F1 scores of 98.42% and 99.50%, respectively, highlighting the model's ability to extract realistic and diagnostically relevant NI-fECG signals.

In 2024, Yang et al. (102) proposed a CycleGAN-based model which utilizes a combined CNN–BiLSTM (Long Short-Term Memory) architecture to improve temporal and morphological fidelity in the generated fECG signal. The approach integrates two generators and two discriminators within the CycleGAN framework: one generator maps abdominal ECG to fECG, and the second maps fECG back to abdominal ECG, ensuring cycle-consistency. The CNN component extracts spatial features from the input, while the BiLSTM captures temporal dependencies, enhancing the model's ability to preserve the waveform structure. The architecture incorporates a hybrid loss function composed of adversarial loss, cycle-consistency loss, and identity loss to optimize both realism and fidelity of the extracted fECG. The approach is evaluated on Dataset 2 and Dataset 9, outperforming the GAN based model proposed by Basak et al. (99) and other AI approaches (80), (89), (108).

##### 3.3.1.3 Hybrid models

They represent a mixture between deep learning and classical signal processing or machine learning techniques. Baldazzi et al. (67) propose a hybrid Signal Quality Assessment (SQA) method that classifies ADSs segments into informative and non-informative using supervised machine learning. Frequency-domain, time-domain, and signal-to-noise ratio (SNR) features are computed and fed into a Random Forest (RF) classifier. Only segments labeled as informative (fetal SNR ≥ 5 dB) are processed further using classical adaptive filtering (QRD-RLS) to extract the fECG. Fetal QRS complexes are detected using standard thresholding, with annotations cross-validated via Doppler signals or manual review. Evaluated on Datasets 6 and 7, the method achieves 88.2% accuracy, 88.4% sensitivity, and an F1-score of 86.5%. While primarily focused on signal quality and QRS detection, this two-stage framework effectively integrates classical filtering and machine learning to improve fetal signal isolation.

Ziani et al. (103) propose a hybrid model that drives fECG extraction by first applying a cascade of signal-processing transforms, Singular Value Decomposition (SVD), Independent Component Analysis (ICA), Continuous Wavelet Transform (CWT), Non-negative Matrix Factorization (NMF) and Empirical Mode Decomposition (EMD), to each single-lead abdominal recording (Dataset 10). These steps produce timescale (TSI) and time–frequency (TFI) image representations in which fetal and maternal ECG components become more distinguishable. A lightweight CNN then classifies each image as “fetal ECG present” or “maternal ECG only.” Its architecture comprises sequential convolutional layers for feature learning, pooling layers for spatial reduction, and fully connected layers for final binary decision. By using the CNN's segment-level predictions to select only fetal-bearing windows for downstream reconstruction the proposed pipeline obtains a 95% accuracy.

##### 3.3.1.4 Specialized architectures

These type of architectures for NI-fECG extraction are characterized by deeply customized neural models designed specifically to address the unique challenges of processing ADSs. Unlike standard neural networks, these models integrate advanced mechanisms—such as transformer encoders, residual U-Nets, graph attention modules, and dual-stream encoders—tailored to enhance the separation of maternal and fetal cardiac signals, improve noise robustness, and preserve diagnostic morphology.

Wang et al. (89) proposed the Canonical-Structured Graph Sparse Attention Network (CSGSA-Net), a graph-based deep learning model that integrates spatial and channel-wise attention mechanisms for precise fECG estimation from ADSs. The architecture processes ADSs using a ResNet19 backbone to extract multi-scale features, followed by the Canonical Spatial Graph Sparse Attention (CSGSA) and Canonical Channel Graph Sparse Attention (CCGSA) modules. These graph-based attention mechanisms enable robust modeling of waveform-level relationships and inter-channel dependencies, crucial for resolving overlapping maternal and fetal signals. Evaluated on both Dataset 9 and the B2 subset of Dataset 11, the model achieved 99.44% accuracy in fetal QRS detection and preserved morphology with MSE = 0.057 and MAE = 0.127.

Rasti et al. (77) introduced a two-stage Res-UNet framework for single-channel fECG extraction. The first Res-UNet estimates the maternal ECG signal, which is then subtracted from the input to obtain a residual. This residual is processed by a second Res-UNet to reconstruct the fetal ECG. Each Res-UNet employs five levels of 1D convolution with skip connections, enabling efficient encoding and reconstruction of signal features while maintaining morphological fidelity. Validated on Datasets 1 and 8, this architecture reported 93.52% accuracy for fetal QRS detection and demonstrated strong morphological conservation, with R² near 1 at high SNR levels, indicating high waveform reconstruction quality.

Lee et al. (105) developed a customized W-net architecture for simultaneous extraction of maternal and fetal ECG signals from single-channel ADSs. The model includes dual encoders and decoders—one for mECG and the other for fECG—using different kernel sizes (35 for broad mECG features and 4 for high-frequency fECG features). Feature subtraction between the encoders enhances fetal-specific pattern learning. Trained on synthetic data (Dataset 2) and tested on real ADSs (Datasets 8 and 9), the model achieved 98.23% accuracy in fetal QRS detection and high PSNR (47.2 dB), confirming its effectiveness in extracting clean fECG signals.

Almadani et al. (107) extended this concept by introducing W-NETR, a W-shaped dual-stream architecture using transformer-enhanced U-Net models (UNETRs) for simultaneous fECG and mECG separation. Each stream includes a transformer-based encoder and a convolutional decoder. The transformer layers employ multi-head self-attention to capture long-range dependencies, while skip connections between encoder and decoder layers preserve spatial and temporal features. Evaluated on Datasets 8 and 9, W-NETR achieved the highest reported accuracy (99.45%) and morphological preservation, with PSNR = 51.8 dB and SSIM = 0.98, outperforming convolution-only architectures in signal quality and robustness.

Rahman et al. (106) presented LinkNet++, an improved variant of LinkNet incorporating dense skip connections and deep supervision to address the low amplitude of fECG signals and their overlap with maternal ECG. The model includes four encoder-decoder blocks with residual ConvBlocks, and feature fusion via dense connections helps mitigate vanishing gradients and preserve signal fidelity across layers. It was trained and evaluated using Leave-One-Subject-Out Cross-Validation on Datasets 9 and 11, yielding 99.6% F1 score and excellent morphology metrics (MAE = 0.068, RMSE = 0.089, PCC = 85.58%), confirming its effectiveness in both waveform reconstruction and fetal QRS detection.

These specialized architectures demonstrate the potential of tailored deep learning designs to significantly improve the quality and diagnostic relevance of extracted fECG signals. Their consistent use of customized modules: graph attention, dual decoders, or transformer encoders, positions them as highly effective for real-world, noisy abdominal recordings where preserving signal morphology is critical.

#### 3.3.2 AI methods for NI-fECG denoising

In NI-fECG, even after the mECG has been subtracted through classical signal-processing methods (e.g., ICA, Event Synchronous Canceller, EMD, Hilbert Huang Transform etc), the extracted fECG often remains contaminated by residual maternal activity that impairs the extraction of diagnostic information. In the following paragraphs we present the identified studies that use AI architectures for NI-fECG denoising, grouped per the categories of AI approaches we have defined in Section V.

##### 3.3.2.1 Neural network based

To address this, Fotiadou et al. (79) proposed a convolutional encoder–decoder network for single-channel fECG denoising. The model processes 1,920-sample segments (∼3.8 s) through eight convolutional and transposed-convolution layers with skip connections to preserve wave morphology. Evaluated on simulated (Datasets 3, 4) and real signals (Dataset 9), it showed strong diagnostic performance: for PR and QT intervals, RMSE = 9.9 ms and 14 ms; R^2^ = 0.77 and 0.81, respectively, with further improvement after excluding extreme values. However, at low SNRs, the model introduced false P/T waves and missed QRS complexes.

An extension to multi-lead input (94) applied the same architecture across four channels with inter-channel skip connections. Tested on Datasets 3, 4 (simulated) and Datasets 9, 16 (real), it achieved ≈13 dB SNR gain for simulated inputs (−20 to 20 dB) and on real data: PCC = 0.87, MASE = 62.1 µV^2^, MAE = 5.4 µV, with a 6.4 dB SNR improvement.

##### 3.3.2.2 Generative AI

More recently, Mvuh et al. (100) demonstrated that generative modeling can push denoising even further under extreme noise conditions. Their conditional GAN framework treats the pre-extracted fECG as the generator's input and learns to produce artifact-free outputs through a 1D convolutional “generator” network enhanced with residual links and dilated convolutions for long-range context. A lightweight 1D PatchGAN discriminator, operating on 70-sample windows, enforces high-frequency realism. Training combines adversarial loss with an MSE reconstruction term, encouraging the generator both to fool the discriminator and to stay close to the ground truth. When applied to synthetic ADSs with input SNRs as low as −20 dB (Dataset 2) and validated on real single-lead fECG (Dataset 9), this adversarial autoencoder achieved an average SNR gain of 20 dB and doubled true QRS detections while halving false positives compared with conventional denoisers (see Table 6).

**Table 6:** Classification, characteristics and performances of the AI models used for NI-fECG denoising.

Included Study	Dataset	Input data characteristics	Performance evaluation	
			R-peak detection	Morphology conservation
Neural Network based models				
Fotiadou et al. (79)	Dataset 3 Dataset 4 Dataset 9	− Segments of 1,920 samples. − Training: 840,000 segments − Testing: 200,000 segments.	Not mentioned	Dataset 9: RMSE 9.9 ms and R^2^ 0.77 for PR interval, RMSE 14 ms and R^2^ 0.81
Fotiadou et al. (94)	Dataset 3 Dataset 4 Dataset 9 Dataset 17	− Segments of 1,920 samples. − Training: 64 segments and − Testing: 64 segments.	Not mentioned	Simulated signals: average $SNR_{imp}$ ≈ 13 dB Real signals:: PCC = 0.87, MASE = 62.1 µV², MAE = 5.4 µV, $SNR_{imp}$6.4 dB
Generative Models				
Mvu et al. (100)	Dataset 2 Dataset 9	− Matrix of 4 × 1,920. − Training: 1,750 simulated ADS segments (Dataset 2); twin pregnancy case is excluded. − Validation: both simulated fECG signals (with different noise levels, that varies from −30 to 0 dB) but also real ADSs signals from Dataset 9 (only one recording, r10).	Dataset 11: SE 67.34%, PPV 78.05%, F1 72.3%	Dataset 2: average $SNR_{imp}$ 4.05 dB

#### 3.3.3 AI methods for diagnosis based on NI-fECG

**Figure 4:**
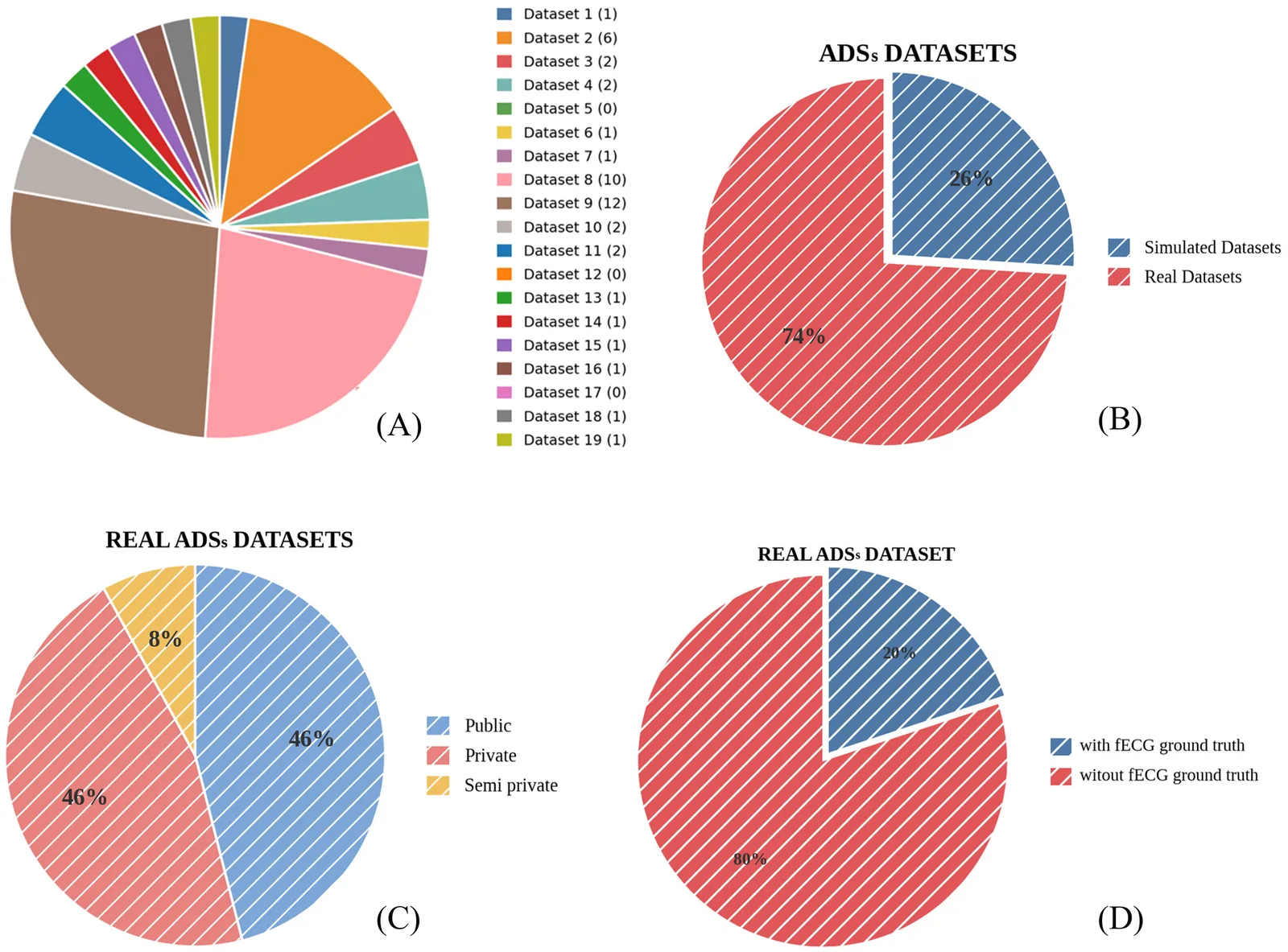
Characteristics of the ADSs datasets: **(A)** the frequency of usage for each dataset, **(B)** the weight of real NI-fECG dataset vs. simulated ones, **(C)** the weights of public, private and semi private datasets form the total number of real NI-fECG datasets, **(D)** the weight of datasets that offers also the ground truth vs. no ground truth from the total real NI-fECG datasets.

Once a clean NI-fECG waveform is obtained through separation from maternal ECG and subsequent denoising, AI can be applied to extract clinically relevant features (QRS complexes, beat locations, intervals and segments durations and amplitudes, morphology features of waves, rhythm abnormalities, etc) and to classify cardiac conditions (Figure 4).

From the studies included in the present review, we have identified few clinical applications ranging from estimation of fetal heart rate (fHR) and its variability to Congenital Heart Disease (CHD) diagnosis, using AI approaches on clean NI-fECG signals (see Table 7).

**Table 7:** Classification, characteristics and performances of the AI models used for diagnosis based on clean NI-fECG.

Included Study	Dataset	Input data characteristics	AI task	Performance evaluation
Neural Network based models				
Darmawahyuni et al. (98)	Dataset 8	− Input data: fECG segments. − Training set: 50 fECG recordings. − Validation: 13 fECG recordings. − Testing set: 5 fECG recordings. − Input data balance: 317,000 segments are labeled as QRS complexes and 126,800 as non-fetal QRS complexes	fHR and variability estimation	Acc = 100%, SE = 100%, SP = 100%, PPV = 100%, F1-score = 100%
Vadivu et al. (96)	Dataset 15	− Input data: images (28 × 28). − Training set: 39 signals. − Test: 11 signals. − Validation set: 5 signals (5-fold cross-validation)	Fetal QRS detection in clean fECG recordings	Acc = 99.46%, SE = 98%, PPV = 97.5%, F1-score = 97.5%
Vries et al. (90)	Dataset 19	fECG segments. Dataset is split into 10 groups (balanced proportion of CHD and healthy cases). 10-fold cross-validation strategy is employed for training. 122 recordings (including both CHD and healthy cases) are set aside for final testing.	Congenital heart disease screening	Acc = 71%, SE = 63% for all CHD, SE = 75% for critical CHD requiring neonatal intervention, SP = 77% specificity
Nakatani et al. (95)	Dataset 16	− Input data: 3s fECG segments − Labels: normal or arrhythmic. − 26 subjects (12 arrhythmic and 14 healthy. − 26-fold cross-validation	Fetal arrythmias	Acc = 96.2%, SE = 100%, SP = 100%,
Wahbah et al. (104)	Dataset 20	− 1 × 1,000 samples ADSs segments. − 5 fold cross-validation	fHR and variability estimation	Acc 93%, F1 0.96
Specialized Architectures				
Fotiadou et al. (108)	Dataset 8. Dataset 14	− Input data: matrix 4 × 30,000 samples (4 channels of clean fECG signals, 1-minute segments, fs 500 Hz). − Training:600 one-minute fECG segments. − Test set: 120 one-minute fECG segments.	fHR Estimation	MSE = 49.4 bpm2, MAE = 2 bpm, PPA = 97.3%, coverage 87.9%

##### 3.3.3.1 Neural network based

Darmawahyuni et al. (98) proposed a lightweight CNN–BiLSTM hybrid that processes 1-s ADSs segments (Dataset 8), with the CNN (8 filters, kernel 3) extracting morphological cues and the BiLSTM (64 units) capturing beat timing. This architecture achieved 100% sensitivity, precision, and F1 (see Table 7) on 68 real ADSs, enabling accurate real-time fHR monitoring without post-detection filtering.

Building on this, Wahbah et al. (104) also adopted a BiLSTM-based framework, using three stacked layers (100, 50, 20 units) with dropout and a weighted classification scheme to address class imbalance. Their addition of an fECG post-processing step effectively reduced false detections, improving accuracy from 93.0% to 94.2% and F1-score from 0.96 to 0.97.

Vadivu and Kavithaa (96) approached fQRS detection from pre-extracted fECG waveforms (Dataset 14; Table 3), adapting LeNet-5 to classify morphological fusion images (28 × 28) combining heartbeat intervals and ECG features. On Dataset 16, their model achieved 99.46% accuracy, 98.00% sensitivity, 97.00% precision, and 97.50% F1 (Table 7), showing strong performance in arrhythmia-oriented fQRS identification.

Moving beyond QRS detection, de Vries et al. (89) investigated AI's potential to augment congenital heart disease (CHD) screening by training a feed-forward network on six-lead fECG from 451 pregnancies (140 CHD cases). Classifying 30-s segments and aggregating probabilities via Bayesian updating, the model achieved 63% sensitivity for all CHD, 75% for critical CHD, 77% specificity, and 71% accuracy, outperforming mid-trimester ultrasound detection rates, though specificity and interpretability remain as limitations.

Finally, Nakatani et al. (94) demonstrated that clean fECG can also enable beat-level arrhythmia classification. Their 1-D CNN processes 3-s fECG windows, learning morphology via convolution-ReLU-pooling layers and classifying normal vs. arrhythmic beats. On a private cohort of 26 subjects, the model achieved 96.2% accuracy, 100% specificity, and 100% recall, surpassing traditional R–R interval methods.

##### 3.3.3.2 Specialized architectures

Fotiadou et al. (108) proposed a specialized architecture for direct fHR estimation from ADSs, bypassing traditional R-peak detection. After removing the maternal ECG via classical subtraction, four channels of NI-fECG (Dataset 8 and Dataset 14) are processed in 1-minute segments by a Dilated Inception CNN (DICNN) with dilation rates of 1, 4, and 8 to capture multi-scale morphology. A 512-unit LSTM models beat-to-beat dependencies, followed by fully connected layers producing 240 fHR estimates per segment (4 Hz). A CNN-based quality assessment filters unreliable windows. On Dataset 14, this DICNN–LSTM pipeline achieved MSE = 49.4 bpm², MAE = 2 bpm, PPA (Percent Positive Agreement) = 97.3%, and coverage = 87.9%, indicating robust continuous fHR tracking.

Collectively, these classification and regression approaches show that once a clean fECG is obtained, AI can accurately extract beat-level timing, detect arrhythmias with >99% F1, and identify complex pathologies with sensitivities approaching those of specialized imaging—highlighting the diagnostic potential of NI-fECG powered by deep learning.

## 4 Discussions

As shown in the Introduction section, NI-fECG is not yet adopted in clinical practice, mainly because extracting a clean signal from ADSs, while preserving diagnostic information and significantly improving SNR, remains a major challenge. To date, most extraction methods rely on classical signal processing, with relatively few studies applying deep learning. A key advantage of deep learning is its ability to automatically learn complex relationships between fECG and mECG, as well as the variable morphology of fECG, without human supervision.

As detailed in the previous section, the studies in this review were grouped according to the main contribution of their AI methods to the NI-fECG processing and analysis pipeline (Figure 3): i) extraction of fECG from ADSs, ii) denoising after extraction, and iii) diagnostic use of NI-fECG.

The following discussion addresses the research questions defined in our Methodology section.

### 4.1 RQ1: what are the important characteristics of the fECG datasets used for AI approaches?

Current AI approaches for NI-fECG rely on a limited number of heterogeneous datasets, where simulated data support algorithm development and objective evaluation, while real datasets remain insufficiently diverse and standardized to ensure robust clinical generalization. The analysis of the reviewed studies reveals that the availability and characteristics of NI-fECG datasets remain one of the major factors influencing the development, evaluation, and generalizability of AI-based approaches. Across the identified studies, both simulated and real ADS datasets play complementary roles.

Simulated ADS datasets account for 26% of those reported in the literature (Figure 4B) and were used in 40% of the reviewed studies (77–80, 94, 97, 100, 102, 104, 105), mainly for training and quantitative benchmarking under controlled SNR, motion-artifact, and different pathology conditions. Conversely, real datasets expose AI models to the physiological variability and complexity of pregnancies and recording artefacts encountered in clinical practice, making them indispensable for assessing clinical applicability. Thus, performance obtained on synthetic data alone should not be assumed to transfer to clinical recordings.

The reviewed literature demonstrates a clear dependence on a relatively small number of benchmark datasets. Although 15 real datasets are identified (Table 3), 16 of the 22 included studies (≈73%) use Dataset 8 and/or Dataset 9 (Figure 4A) for model development and evaluation. This concentration around a few publicly available databases eases cross-study comparison studies but also introduces the risk of dataset-specific optimization, where models become highly adapted to the characteristics of a small number of recording protocols rather than learning representations that generalize across different clinical environments. A related and underappreciated issue is that the large sample counts reported by many deep-learning studies (tens of thousands of training segments) are drawn from a handful of subjects within these same datasets. These segments are highly correlated rather than statistically independent observations, which likely inflates the reported AI model performance and limits confidence in the reported generalization capabilities. Furthermore, only a small proportion (20%, Figure 4D) of available real datasets include invasive direct fetal ECG recordings that can serve as reliable ground truth for evaluating waveform reconstruction and morphology preservation.

Another important observation concerns the heterogeneity of the available datasets. Considerable differences exist in gestational age (GS), recording duration, sampling frequency, number and placement of abdominal electrodes, acquisition hardware, preprocessing protocols, and annotation procedures. While this diversity reflects real clinical variability, it also makes direct comparison between AI models challenging because reported performance may depend as much on dataset characteristics as on the proposed algorithm itself. The absence of standardized acquisition protocols and benchmarking methodologies therefore remains a significant obstacle to objective performance comparison across studies.

Overall, the evidence indicates that the main bottleneck for further progress is no longer AI architecture design in isolation, but the availability of larger, standardized, and clinically representative real datasets, together with benchmarking protocols that allow fair comparison across independent cohorts.

### 4.2 RQ2: do AI approaches improve the outcome of the NI-fECG processing and analysis pipeline?

The studies included in this review consistently show that AI improves NI-fECG processing and analysis. Unlike conventional techniques that rely on predefined filtering rules or statistical assumptions, AI models learn nonlinear maternal-fetal signal relationships directly from data, yielding more accurate extraction, better noise suppression, and more reliable automated analysis. expanding NI-fECG's role from fetal heart-rate estimation toward comprehensive cardiac assessment.

The greatest advances are in fetal ECG extraction, where deep learning has largely replaced adaptive filtering and blind source separation. CNN/RNN models [Zhong et al. (80); Vo et al. 93] achieve high sensitivity and precision by learning spatiotemporal dependencies directly from abdominal recordings; specialized architectures such as W-Net (105) and W-NETR (107) simultaneously estimate maternal and fetal ECG while preserving morphology; generative approaches such as Basak et al.'s 1D CycleGAN (99) optimize waveform realism via adversarial and cycle-consistency losses rather than detection accuracy alone; and hybrid models such as Ghonchi et al.'s dual-attention autoencoder (109) combine deep learning with signal-processing principles for added robustness.

However, the evidence synthesized in this review also suggests that the reported superiority of AI methods should be interpreted with caution. Metrics exceeding 99% (89, 98, 105, 106) are predominantly obtained on the same two benchmarks discussed in the answer for RQ1, with studies differing in preprocessing, validation strategy, and metrics, which limits direct comparison. Thus, while AI's advantage over classical signal processing is well supported, the evidence does not support any one architecture being universally superior (a more detailed discussion under RQ3).

A further finding of this review is that the objectives of AI research in this field have gradually evolved. Early studies primarily aimed to improve fetal QRS detection and fetal heart rate estimation, whereas more recent work increasingly focuses on preserving the complete fetal ECG waveform to enable clinically meaningful diagnosis. This shift is particularly evident in the generative and specialized architectures noted above, which explicitly optimize signal morphology rather than only detection accuracy. Accurate R-peak detection secures fetal heart rate but not the features of P wave, ST segment, QT interval, and T wave, the parameters needed to identify fetal hypoxia, long QT syndrome, arrhythmias, etc. Morphology preservation should therefore be a primary evaluation criterion, not a complementary one. However, only 11 of the 16 extraction/denoising studies evaluated it at all.

AI has also extended beyond extraction to automated diagnosis: six studies applied it to fHR estimation, fQRS detection, arrhythmia detection, and CHD classification, consistently improving automation and reducing observer dependence relative to rule-based methods. This diagnostic use remains considerably less mature than extraction, so more evidence is needed before routine obstetric integration.

Overall, AI has clearly transformed the technical performance of the NI-fECG pipeline, extraction, denoising, and automated analysis alike, but that evidence base remains bounded by dependence on a handful of benchmark datasets, heterogeneous evaluation methods, and limited morphology assessment. Progress from here depends less on further architectural complexity than on standardized benchmarking, multicenter validation, morphology-aware evaluation, and prospective clinical testing.

### 4.3 RQ3: which AI model is the most appropriate to process and analyze the NI-fECG signal for medical decision- making?

The reviewed literature demonstrates that no single AI architecture is universally optimal for all stages of the NI-fECG processing and analysis pipeline. Instead, the suitability of an AI approach depends on the specific objective, whether accurate fetal ECG extraction, morphology preservation, denoising, or automated diagnosis.

Neural network-based models [CNNs for spatial features, LSTMs/BiLSTMs for temporal dependencies, e.g., Zhong et al. (80), Vo et al. 93] remain the most widely adopted across all three pipeline stages, reflecting their maturity as a robust general-purpose baseline. Generative models are a more recent addition, extending AI beyond fQRS detection toward realistic waveform reconstruction: Basak et al.'s CycleGAN (99) and Yang et al.'s CNN–BiLSTM CycleGAN (102) both explicitly optimize morphology via adversarial learning, though generative models remain comparatively underused and have not yet been applied to diagnostic classification tasks. Specialized architectures purpose-built for NI-fECG [W-Net (105), W-NETR (107), LinkNet++ (106), CSGSA-Net 89] and hybrid models combining deep learning with classical signal processing [Baldazzi et al. (67), Ghonchi et al. 109] report among the highest metrics, suggesting domain-specific design is currently the most promising direction for extraction performance.

However, this apparent superiority should not be read as settled. The highest-performing specialized architectures are evaluated on a limited set of benchmark datasets under differing preprocessing, splits, and metrics, thus their margin over conventional CNN/RNN baselines cannot be confidently attributed to architecture alone rather than to evaluation conditions. Few studies report external validation on independent cohorts or provide uncertainty estimates, further limiting confidence in generalization. The evidence therefore does not support ranking architectures by performance, but it supports choosing architecture according to the intended application, available data, and required interpretability, consistent with the shift toward morphology-aware, clinically oriented design already noted under RQ2.

Overall, neural network-based models offer the most mature and broadly applicable solution; specialized architectures currently report the highest extraction performance, though on the narrowest evidence base; and generative models offer a promising but underexploited route to morphology preservation. Future work should prioritize generalization, computational efficiency, explainability, and standardized cross-dataset validation over further architectural complexity, since these are more likely to determine clinical translation than marginal gains on benchmark metrics.

### 4.4 RQ4: what are the current challenges of AI models for processing the NI-fECG signal

Although the reviewed studies show that AI has substantially advanced NI-fECG processing, several challenges continue to limit its translation into routine clinical practice. Consistent with the evidence discussed under RQ1–RQ3, these limitations are no longer primarily about whether AI can extract a fetal ECG signal, but about dataset representativeness, standardized evaluation, and clinical validation.

The most persistent challenge is dataset availability. As noted under RQ1, evaluation remains concentrated on the same two real datasets, Dataset 8 and Dataset 9, which eases comparison but risks overfitting to their specific acquisition characteristics rather than to genuinely generalizable representations. A related but distinct limitation is ground-truth scarcity: only 3 of the 15 real datasets identified in this review (20%) include a direct fECG reference, since direct recording requires an invasive scalp electrode available only during labor. Larger, multicenter datasets, covering diverse gestational ages, maternal body mass indices, fetal positions, pathological pregnancies, and acquisition protocols, remain the clearest path for improving generalization, more so than further architectural refinement. Addressing them is what will turn promising prototypes into reliable diagnostic tools.

A second challenge is the absence of standardized benchmarking. Differing preprocessing pipelines, segmentation strategies, validation protocols, and metrics make direct comparison between architectures difficult, and this heterogeneity, rather than genuine architectural superiority, likely explains why models built on entirely different principles (RQ3) report similarly high, near-99% performance: without a shared benchmark, that convergence cannot be distinguished from an artifact of favorable evaluation conditions. Compounding this, evaluation still centers on fetal QRS detection: only 11 of the 16 extraction and denoising studies assess waveform morphology preservation, even though diagnosis depends on the full ECG waveform rather than R-peak timing alone. Morphology-aware metrics should become a standard, not optional, component of any future benchmarking framework.

Regarding the AI architecture used, the field is trending toward increasing sophistication, attention mechanisms, transformers, GANs, hybrid frameworks, but this review's evidence does not support complexity alone as the path to clinical adoption. Most of these models remain black boxes, offering little insight into which signal features drove a given decision. In a safety-critical context like fetal monitoring, this limits clinician trust and complicates approval by regulators such as the U.S. Food and Drug Administration (FDA) and European Medicines Agency (EMA), which require interpretable mechanisms for medical devices. Explainable AI methods that surface the waveform features behind a decision would address both concerns at once.

A related and, arguably, more consequential gap is between technical and clinical validation. Nearly all reviewed models are evaluated retrospectively, offline, on curated datasets; prospective validation under real obstetric conditions, maternal movement, shifting fetal position, poor electrode contact, rare cardiac pathologies, is essentially absent from this literature. It therefore remains unknown how these models behave outside the conditions under which they were trained and tested. Future work should evaluate not only algorithmic accuracy but clinical outcomes, workflow integration, and user acceptance.

**Figure 5:**
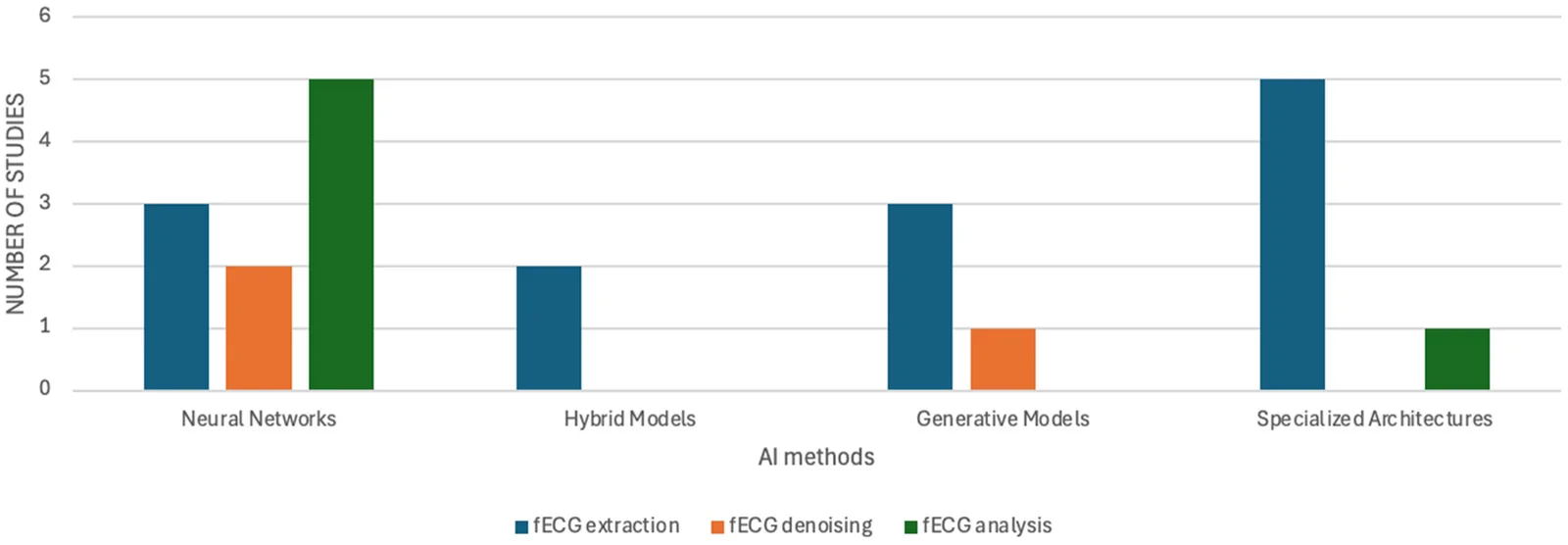
The number of studies per contribution to the fECG processing and analysis pipeline and per type of AI models.

A further, still largely unexplored, direction is extending AI beyond isolated extraction toward integrated fetal health assessment. As Figure 5 shows, no study in this review combines extraction, denoising, and diagnostic classification within a single end-to-end model, despite the efficiency and error-reduction gains such joint learning could offer. Combining high-fidelity fECG reconstruction with multimodal maternal-fetal data and wearable monitoring could move the field from isolated signal-processing algorithms toward genuine clinical decision-support platforms, though this remains a direction rather than a demonstrated capability in the current literature.

Overall, further progress is likely to depend less on marginal gains in benchmark performance and more on resolving these translational barriers: standardized multicenter datasets, harmonized and morphology-aware benchmarking, explainable and computationally efficient models, and prospective clinical validation. These, rather than additional architectural sophistication, are what would give AI-assisted NI-fECG systems the evidence base needed for routine clinical adoption.

## 5 Conclusions

This review systematically examined the AI architectures proposed for NI-fECG processing and analysis, addressing the characteristics and availability of the datasets used (RQ1), the impact of AI on the extraction–denoising–diagnosis pipeline (RQ2), model suitability for supporting medical decision-making (RQ3), and the challenges that currently limit clinical translation (RQ4). To our knowledge, this is the first systematic review focused exclusively on AI for NI-fECG signal processing and analysis. Across the studies reviewed, AI-based extraction, denoising, and diagnostic classification generally outperform classical signal processing techniques, and an increasing number of models preserve fECG morphology alongside detection accuracy. No single architecture, however, is universally superior; the most appropriate model depends on the specific pipeline stage, the available data, and the interpretability the application requires.

These advances remain constrained by the limitations identified throughout this review: concentrated and limited-diversity datasets, the absence of standardized benchmarking and morphology-aware evaluation, black-box interpretability, and an almost complete lack of prospective clinical validation. Progress toward clinical adoption therefore depends less on further architectural sophistication than on closing these translational gaps, through standardized multicenter datasets, harmonized and morphology-aware benchmarks, explainable models, and validation under real obstetric conditions. Meeting these needs, rather than incremental performance gains, is what would give AI-assisted NI-fECG systems the evidence base required for routine clinical use.

## Data Availability

The original contributions presented in the study are included in the article/Supplementary Material, further inquiries can be directed to the corresponding author/s.
